# Associations of home confinement during COVID-19 lockdown with subsequent health and well-being among UK adults

**DOI:** 10.1007/s12144-022-03001-5

**Published:** 2022-03-15

**Authors:** Koichiro Shiba, Richard G. Cowden, Victor Counted, Tyler J. VanderWeele, Daisy Fancourt

**Affiliations:** 1 Department of Epidemiology, Harvard T.H. Chan School of Public Health, Boston, MA USA; 2 Human Flourishing Program, Institute for Quantitative Social Science, Harvard University, Cambridge, MA USA; 3 School of Psychology and Counselling, College of Health and Behavioral Sciences, Regent University, Virginia Beach, VA USA; 4 Department of Biostatistics, Harvard T.H. Chan School of Public Health, Boston, MA USA; 5 Department of Behavioural Science and Health, University College London, London, UK

**Keywords:** COVID-19, Home confinement, Well-being, Mental health, Outcome-wide epidemiology, UK

## Abstract

During the COVID-19 pandemic, the United Kingdom (UK) government introduced public health safety measures to mitigate the spikes in infection rates. This included stay-at-home orders that prevented people from leaving their homes for work or study, except for urgent medical care or buying essential items. This practice could have both short and long-term implications for health and wellbeing of people in the UK. Using longitudinal data of 10,630 UK adults, this study prospectively examined the association between home confinement status during the stringent lockdown in the UK (March 23-May 13, 2020) and 20 indicators of subjective well-being, social well-being, pro-social/altruistic behaviors, psychological distress, and health behaviors assessed approximately one month after the stringent lockdown ended. All analyses adjusted for socio-demographic characteristics and social isolation status in the beginning of the pandemic. Home confinement during the lockdown was associated with greater subsequent compliance with COVID-19 rules, more perceived major stressors, and a lower prevalence of physical activity. There was modest evidence of associations with lower life satisfaction, greater loneliness, greater depressive symptoms, greater anxiety symptoms, and more perceived minor stressors post-lockdown. However, there was little evidence that home confinement was associated with other indices of subsequent health and well-being. While our study shows that home confinement impacts some indices of subsequent health and wellbeing outcomes even after lockdown, the degree of the psychological adaptation to the difficult confinement behavior remains unclear and should be further studied.

**Supplementary Information:**

The online version contains supplementary material available at 10.1007/s12144-022-03001-5.

## Introduction

In response to rapid global transmission of severe acute respiratory syndrome coronavirus 2 (SARS-CoV-2), health authorities in many parts of the world instituted public health control measures to limit transmission of the virus (Counted et al., 2020; Govender et al., 2020). Community mitigation strategies designed to control the spread of SARS-CoV-2 varied across countries, with some enforcing stringent lockdowns at one or more points during the coronavirus disease 2019 (COVID-19) pandemic (Counted et al., 2021). For example, the United Kingdom (UK) government implemented strict lockdown laws on several occasions between March 2020 to March 2021. During the stringent public health control measures, nonessential travel, social gatherings, and public activities were banned or heavily restricted (Ogden, 2020). People were ordered to stay at home unless they needed to purchase essential items, fulfill employment obligations, or undertake essential activities (e.g., healthcare appointments) that could not be conducted remotely (Storr et al., 2021). These directives resulted in many people being emplaced within their homes for extended periods of time (Counted et al., 2021; Devine-Wright et al., 2020)*.*

So far, research suggests that home confinement within the context of the COVID-19 pandemic has negatively affected health and well-being. Findings of cross-sectional studies involving samples of participants observing compulsory stay-at-home orders indicated that home confinement regulations were associated with higher levels of psychological distress, less physical activity, and lower sleep quality (Dönmez et al., 2021; Pinto et al., 2020; Sang et al., 2020). In other cross-sectional research that included a retrospective assessment component to estimate changes in outcomes from before to during the COVID-19 pandemic, many participants reported a decline in mental health (e.g., depression), subjective well-being (e.g., life satisfaction), health (e.g., weight change), health behaviors (e.g., physical activity), and social participation (Ammar et al., 2020; Cellini et al., 2021; Fernandez-Rio et al., 2020). Similar findings have been reported in a few longitudinal studies that tracked facets of well-being while mandatory lockdowns were in effect. For example, a pre-post longitudinal study found significantly worse physical activity, sleep problems, and self-perceived well-being among Spanish adults after they were confined to their homes during the lockdown that was imposed (Martínez-de-Quel et al., 2021). In a prospective study conducted in three European countries (Belgium, Hungary, and Spain), (Simor et al., 2021) reported that disrupted or poorer sleep quality due to time spent confined at home was associated with an increase in negative psychological (e.g., depression) and physical (e.g., somatic complaints) symptoms the following day.

Taken together, existing research has contributed to enhancing our understanding of how increased home confinement during the COVID-19 pandemic affected health and well-being. However, there are several unresolved gaps in this body of knowledge. First, most findings are based on cross-sectional data, which are insufficient for establishing causality (VanderWeele, 2021). Even if retrospectively reported survey items are used to mitigate concerns about reverse causality, bias and contamination can be introduced when participants are queried under stressful circumstances like the COVID-19 pandemic (Simor et al., 2021). Hence, more longitudinal studies are needed to better understand the implications of home confinement for health and well-being. Second, prior research has tended to infer home confinement by the severity of lockdown regulations implemented in a particular place and point in time, potentially misclassifying the home the confinement behavior of individuals who did not adhere to such regulations. To estimate the impact of home confinement on health and well-being more rigorously, it is important to directly assess individuals' actual home confinement behavior. Third, previous research has generally reported on a single or narrow set of outcomes, providing a limited account of how home confinement might be associated with health and well-being more broadly. Fourth, prior studies have focused on the implications of home confinement for health and well-being *within* periods of lockdown. Research is needed to determine whether there are longer-term consequences of home confinement *after* lockdown restrictions have been lifted, which could provide insight into potential lingering effects of home confinement and inform public health initiatives to support those confined to their homes during lockdowns.

The current study fills the abovementioned knowledge gaps by using prospective data from a large sample of UK adults to estimate potential causal effects of home confinement during the COVID-19 pandemic on a wide range of health and well-being outcomes. More specifically, we examine associations between home confinement during the initial period of stringent lockdown in the UK (March 23—May 13, 2020) and indices of psychological distress, subjective well-being, social well-being, prosocial/altruistic behaviors, and health behaviors assessed approximately one month after the lockdown ended.

## Methods

### Data

We used data from the UCL COVID-19 Social Study, a large prospective panel study on the psychosocial experiences of UK adults ($\ge$ 18 years old) during the COVID-19 pandemic. Three primary recruitment approaches were used. First, convenience sampling was used, including promoting the study through existing networks and mailing lists (including large databases of adults who had previously consented to be involved in health research across the UK), print and digital media coverage, and social media. Second, more targeted recruitment was undertaken focusing on groups who were anticipated to be less likely to take part in the research via our first strategy, including (i) individuals from a low-income background, (ii) individuals with no or few educational qualifications, and (iii) individuals who were unemployed. Third, the study was promoted via partnerships with third sector organisations to vulnerable groups, including adults with pre-existing mental health conditions, older adults, carers, and people experiencing domestic violence or abuse. Recruitment was refreshed in August when participants who were lost-to-follow-up were recontacted. To account for the nonrandom nature of the sampling design, all data were weighted to the proportions of gender, age, ethnicity, education, and country of living obtained from the Office for National Statistics. Further details on sampling and weighting are available elsewhere in the User Guide https://osf.io/jm8ra/ (CSSUserGuide, 2021; Fancourt et al., 2021).

**Fig. 1:**
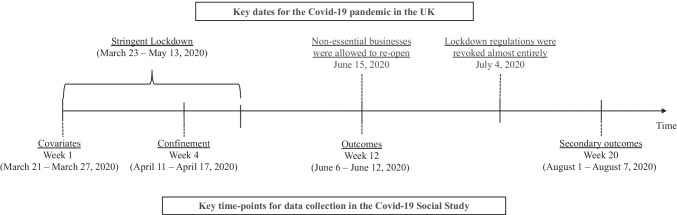
Timelines of Data Collection and Key Dates for the Covid-19 Pandemic in the UK

The study began on March 21, 2020, and data were collected weekly from online participants. For this analysis, we used data from the participants who were recruited from March 21 and March 27, 2020 (week 1: *n* = 28,847). Of those, 16,758 individuals completed the follow-up questionnaire administered in week 4 (April 11—April 17, 2020), which assessed home confinement status during the stringent lockdown in the UK (March 23—May 13, 2020). We excluded those who did not participate in the week 12 survey (June 6—June 12, 2020), from which the outcome data were drawn (*n* = 6,128). The final analytic sample consisted of 10,630 individuals. Figure 1 illustrates the timeline of data collection and key dates, including the three time points that we used for our main analysis (i.e., week 1, week 4, and week 12), for the COVID-19 pandemic in the UK. The UCL Research Ethics Committee approved the study, and all participants provided electronic informed consent.

### *Measures*

#### Home Confinement During the Lockdown

In week 4, participants reported their confinement status during the lockdown in response to the question, “What is your current isolation status?” from the options: 1 = “I am in full isolation, not leaving my home at all”, 2 = “I am staying at home, only leaving for exercise, food shopping, accessing medication, or essential activity permitted by government guidelines”, 3 = “I am staying at home, only leaving for exercise, food shopping or accessing medication AND work OR other essential task (e.g. volunteering)”, 4 = “I am NOT following the stay-at-home recommendations but am adhering to social distancing when in public (e.g. staying 2 m away from others)”, 5 = “I am NOT following the stay-at-home recommendations or social distancing when I am out”, 6 = “I am leaving the house for more reasons than those listed above but am adhering to social distancing in public (e.g., staying 2 m away from others)”, and 7 = “I am leaving the house for more reasons than those listed above and am NOT adhering to social distancing in public (e.g., staying 2 m away from others)”. We defined option 1 (“I am in full isolation, not leaving my home at all”) as home confinement; those who selected the other options were defined as not being confined.

#### Outcomes

To examine the persistent impacts of home confinement after the stringent lockdown, we used the outcome data from week 12—approximately one month after the stringent lockdown ended. We examined 20 indices of health and well-being as outcomes, including psychological distress (depressive symptoms, anxiety, number of minor stressors, number of major stressors, and thoughts of self-harm), subjective well-being (life satisfaction, happiness, and meaning), social well-being (social support and loneliness), prosocial/altruistic behaviors (volunteering, caring, and subsequent compliance with COVID-19 rules), and health behaviors (unhealthy change in smoking, unhealthy change in alcohol drinking, unhealthy change in diet, gentle physical activity, high-intensity physical activity, exercising at home, and good sleep). We chose these outcomes because they represent distinct domains of human well-being (VanderWeele, 2017). Supplementary Table S1 provides further details about the measurement of each outcome (e.g., specific item wording or names of validated scales used to assess the outcomes and how we operationalized the variables).

#### Covariates

All covariates were taken from week 1 (March 21—March 27, 2020), around the time when the strict lockdown in the UK was initiated on March 23, 2020. These covariates included sociodemographic characteristics (age, gender, race, living alone, education, employment, any key worker role, and low income), health conditions and health behaviors (number of health conditions, current smoking, and number of alcohol drinks in the past week, current smoking status, and number of alcoholic drinks in the past week), pre-pandemic religious service attendance, social relationships (frequency of meeting up with people in usual life and number of close friends), personality (neuroticism, extraversion, openness, agreeableness, and conscientiousness), and isolation status in week 1. Isolation status in week 1 was binary and defined as whether or not respondents were “staying at home” (not leaving the house for everything apart from exercise, shopping for essentials, or medical need). Adjusting for the isolation status in week 1 as a proxy for pre-baseline level of the exposure (i.e., home confinement) can help rule out reverse causation (health and well-being leading to home confinement) and some unmeasured confounding (VanderWeele et al., 2020).

### Statistical Analysis

We used an outcome-wide analytic approach, which enables a holistic assessment of the impact of a single exposure on a wide range of outcomes and has several methodological advantages (e.g., being less susceptible to p-hacking and publication bias) (VanderWeele et al., 2020). We used separate regression models to regress each outcome on home confinement during the lockdown, adjusting for the pre-baseline covariates, including isolation status as a proxy for pre-baseline home confinement status, measured at week 1. We used a different model depending on the type of outcome: (1) linear regression for continuous outcomes (life satisfaction, happiness, meaning, social support, loneliness, compliance with COVID-19 rules, depressive symptoms, anxiety, and the number of minor and major stressors), (2) modified Poisson regression with robust variance estimation for non-rare binary outcomes with a prevalence of $\ge$ 10% (caring, no unhealthy change in drinking, no unhealthy change in diet, gentle physical activity, exercising at home, and good sleep) (Zou, 2004), and (3) logistic regression for rare binary outcomes with a prevalence of < 10% (volunteering, thoughts of self-harm, no unhealthy change in smoking behaviors, and high-intensity physical activity). All continuous outcomes were standardized (mean = 0, standard deviation = 1), so the effect estimates can be interpreted in terms of a standard deviation change in the outcome variable. Modified Poisson regression models for nonrare binary outcomes estimate risk ratios, and logistic regression models for rare binary outcomes estimate odds ratios approximating risk ratios. We used Bonferroni correction to account for multiple testing.

We performed four sensitivity analyses. First, to evaluate the robustness of our effect estimates to unmeasured confounding, we calculated E-values for each exposure-outcome association (VanderWeele & Ding, 2017). E-values quantify the minimum strength of association on the risk ratio scale that an unmeasured confounder would need to have with both the exposure and outcome, above and beyond the adjusted covariates, to explain away the observed association. Second, we examined whether the associations between home confinement and the outcomes differ by the motivation for home confinement. Specifically, we estimated the associations with the outcomes for confinement because of being high-risk and confinement for reasons other than being high-risk, respectively. Third, because home confinement was not an available option for most key workers (e.g., healthcare workers) and estimating the impacts of home confinement for them may not be meaningful, we excluded key workers from the analysis and examined the same associations among the remaining sample. Fourth, to examine whether the associations with home confinement during the lockdown are robust to the timing of outcome assessment, we used outcomes from week 20 (August 1—August 7, 2020; approximately one month after the lockdown regulations were revoked almost entirely).

Using the *mice* package in R, we conducted multiple imputation by chained equations to impute missing data on all variables (van Buuren & Groothuis-Oudshoorn, 2010). Supplementary Table S2 shows the amount of data missing in the analytic sample. After generating five imputed datasets, we performed the above analyses using each imputed dataset and combined the results across imputations based on Rubin’s rule (Rubin, 2004). All analyses were conducted in R, version 3.6.0.

## Results

Table 1 shows the characteristics of the weighted study sample in week 1 according to the home confinement status in week 4. Of the analytic sample (*n* = 10,630), 1,458 individuals (13.7%) reported home confinement in week 4. Compared to those with no home confinement (*n* = 9,172), individuals with home confinement were more likely to report lower educational attainment (e.g., General Certificate of Secondary Education or below: 45% vs. 31% in the non-confined group), low-income status (< £30,000: 65% vs. 46%), more health conditions (mean count = 1.84 vs. 0.81), and staying at home (5.7% vs. 3.0%) in week 1; on the other hand, the same confined group was less likely to report employment status (31% vs. 60%) and any key worker role (8.5% vs. 22%).

**Table 1:** Weighted Sample Characteristics at Baseline by Home Confinement Status During the Stringent Lockdown in the UK, COVID-19 Social Study (*n* = 10,630).^a^

Characteristics at the beginning of lockdown	Home confinement during the stringent lockdown			
	Not confined		Confined	
	n (%)	Mean (SD)	n (%)	Mean (SD)
Total	9,172 (86.3)		1,458 (13.7)	
Sociodemographic factors				
Age, years		48 (16)		54 (17)
Female gender	4,532 (50)		792 (55)	
Non-white ethnicity	1,172 (13)		187 (13)	
Living alone	1,697 (18)		281 (19)	
Education				
GCSE or below	2,818 (31)		658 (45)	
A levels or equivalent	3,098 (34)		506 (35)	
Degree or above	3,256 (36)		294 (20)	
Employed	5,498 (60)		459 (31)	
Any key worker role	1,987 (22)		124 (8.5)	
Low Income (< £30 000)	3,828 (46)		814 (65)	
Physical health and health behaviors				
Number of health conditions		0.81 (1.19)		1.84 (1.62)
Smoking status				
Current smoker	1,009 (11)		195 (13)	
Ex-smoker	2,275 (25)		435 (30)	
Non-smoker	5,888 (64)		828 (57)	
Number of alcoholic drinks in the past week		3.9 (5.3)		2.6 (4.5)
Pre-pandemic service attendance				
At least once a week	439 (5.7)		35 (3.0)	
Less than once a week	1,305 (17)		185 (15)	
Not at all	6,013 (78)		979 (82)	
Staying at home	439 (5.7)		35 (3.0)	
Social relationships				
Meeting up with people in usual life				
Every day	886 (9.7)		138 (9.5)	
Less than once a week	2,845 (31)		513 (35)	
Once a week or more often	5,441 (59)		807 (55)	
Number of close friends		4.6 (3.2)		3.9 (2.9)
Personality				
Neuroticism (range: 3–21)		11.3 (4.4)		11.9 (4.7)
Extraversion (range: 3–21)		12.5 (4.3)		12.2 (4.3)
Openness (range: 3–21)		14.7 (3.3)		14.5 (3.4)
Agreeableness (range: 3–21)		15.3 (3.1)		15.6 (3.2)
Conscientiousness (range: 3–21)		15.7 (3.00)		15.7 (3.3)

Abbreviations: SD, standard deviation

^a^ Confinement during the stringent lockdown (March 23—May 13, 2020) was assessed in week 4 (April 11—April 17, 2020). Covariates were from the beginning of the stringent lockdown (week 1; March 21—March 27, 2020). Data were weighted to the proportions of gender, age, ethnicity, education, and country of living obtained from the Office for National Statistics

Table 2 shows the estimated beta coefficients (continuous outcomes), risk ratios (nonrare binary outcomes), and odds ratios (rare binary outcomes) for home confinement status during the stringent lockdown. Home confinement in week 4 was associated with increased subsequent compliance with COVID-19 rules post-lockdown (standardized beta = 0.29; 95% confidence interval: 0.18, 0.40), number of major stressors (standardized beta = 0.23; 0.10, 0.36), and lower likelihood of engaging in gentle physical activity (risk ratio = 0.58; 0.51, 0.68) and high intensity physical activity (odds ratio = 0.45; 0.27, 0.74) in week 12. These associations remained below the *p* = 0.05 threshold after accounting for multiple testing via Bonferroni correction. Evidence of associations between home confinement and decreased life satisfaction (standardized beta = -0.11; -0.22, -0.01) as well as increased loneliness (standardized beta = 0.12; 0.02, 0.23), depressive symptoms (standardized beta = 0.13; 0.01, 0.24), anxiety (standardized beta = 0.12; 0.01, 0.22), and number of minor stressors (standardized beta = 0.15; 0.04, 0.26) was more modest; however, none of these associations were below *p* = 0.05 after Bonferroni correction. We found little evidence of associations between home confinement and other health and well-being outcomes assessed in week 12. Point estimates from the subgroup analysis (Supplementary Figure S1 for continuous outcomes and Supplementary Figure S2 for binary outcomes) were generally similar, although effect sizes tended to attenuate when examining those staying at home for reasons other than being high risk and when assessing outcomes in week 20.

**Table 2:** Home Confinement During the Stringent Lockdown and Post-lockdown Health and Well-being in the UK, COVID-19 Social Study (n = 10,630).^a^

Outcomes in week 12	Home confinement during the stringent lockdown			
	Not confined (*n* = 9,172)		Confined (*n* = 1,458)	
	Reference	β^b^^,d^	RR/OR^c,d^	95% CI
Subjective well-being				
Life satisfaction	0.00	-0.11^*^		(-0.22, -0.01)
Happiness	0.00	-0.11		(-0.21, 0.00)
Meaning	0.00	-0.10		(-0.21, 0.01)
Social well-being				
Social support	0.00	0.00		(-0.12, 0.11)
Loneliness	0.00	0.12^*^		(0.02, 0.23)
Prosocial/altruistic behavior				
Volunteering	1.00		0.64	(0.37, 1.13)
Caring	1.00		0.85	(0.64, 1.14)
Compliance with COVID-19 rules	0.00	0.29^***^		(0.18, 0.40)
Psychological distress				
Depressive symptoms	0.00	0.13^*^		(0.01, 0.24)
Anxiety	0.00	0.12^*^		(0.01, 0.22)
Number of minor stressors	0.00	0.15^**^		(0.04, 0.26)
Number of major stressors	0.00	0.23^***^		(0.10, 0.36)
Thoughts of self-harm	1.00		0.90	(0.42, 1.93)
Health behaviors				
No unhealthy change in smoking	1.00		1.40	(0.75, 2.64)
No unhealthy change in alcohol drinking	1.00		1.00	(0.97, 1.03)
No unhealthy change in diet	1.00		0.99	(0.92, 1.05)
Gentle physical activity	1.00		0.58^***^	(0.51, 0.68)
High intensity physical activity	1.00		0.45^***^	(0.27, 0.74)
Exercising at home	1.00		1.12	(0.94, 1.31)
Good sleep	1.00		1.00	(0.85, 1.17)

Abbreviations: CI, confidence interval; RR, risk ratio; OR, odds ratio

^*^ *p* < 0.05 before Bonferroni correction; ** *p* < 0.01 before Bonferroni correction; *** *p* < 0.05 after Bonferroni correction (the p-value cutoff for Bonferroni correction is *p* = 0.05/20 outcomes = *p* < 0.0025)

^a^ Home confinement during the stringent lockdown (March 23—May 13, 2020) was assessed in week 4 (April 11—April 17, 2020). Outcomes were assessed in week 12 (June 6—June 12, 2020). Covariates were measured at the beginning of the lockdown (week 1, March 21—March 27, 2020). The analytic sample was restricted to those who had participated in the survey in both week 1 and week 12. Multiple imputation was performed to impute missing data on the covariates and the outcomes

^b^ All continuous outcomes (life satisfaction, happiness, meaning, social support, loneliness, compliance with COVID-19 rules, depressive symptoms, anxiety, and number of minor and major stressors) were standardized (mean = 0, standard deviation, 1), and β was the standardized effect size

^c^ The estimates for the outcomes of volunteering, thoughts of self-harm, no unhealthy change in smoking behaviors, and high intensity physical activity were odds ratios estimated via weighted logistic regression; these outcomes were rare (prevalence < 10%), so the odds ratios would approximate the risk ratios. The estimates for other nonrare, dichotomized outcomes (caring, no unhealthy change in drinking, no unhealthy change in diet, gentle physical activity, exercising at home, and good sleep) were risk ratios estimated via weighted Poisson regression

^d^ All models were controlled for pre-baseline participants’ characteristics from week 1, including sociodemographic characteristics (age, gender, race, living alone, education, employment, any key worker role, and low income), health conditions and health behaviors (number of health conditions, current smoking, and number of alcohol drinks in the past week, current smoking status, and number of alcoholic drinks in the past week), pre-pandemic religious service attendance, social relationships (frequency of meeting up with people in usual life and number of close friends), personality (neuroticism, extraversion, openness, agreeableness, and conscientiousness), and the pre-baseline exposure level (home confinement at week 1). Data were weighted to the proportions of gender, age, ethnicity, education, and country of living obtained from the Office for National Statistics

The calculated E-values (Table 3) suggested that some observed associations between home confinement and subsequent well-being might be moderately robust to unmeasured confounding. For example, for the association between home confinement and number of major stressors (standardized beta = 0.23), an unmeasured confounder that was associated with both the exposure and outcome—above and beyond the adjusted covariates—by risk ratios of 1.77 each could fully explain away the observed association, but weaker joint confounder associations could not; and confounder risk ratio associations of 1.40-fold each could shift the confidence interval to include the null, but weaker confounder associations could not. As shown in Supplementary Table S3, the conditional associations of the observed covariates with outcomes were generally weaker than the magnitudes suggested by the E-values, even for covariates with particularly strong associations with an outcome. For example, the risk ratio for the conditional association between the number of major stressors and its strongest predictor—non-white ethnicity—was 1.27, whereas the E-value for home confinement was 1.77.

**Table 3:** Robustness to Unmeasured Confounding (E-Values) of Associations Between Home Confinement During the Stringent Lockdown and Post-lockdown Health and Well-being in the UK, COVID-19 Social Study (*n* = 10,630)

Outcomes in week 20	Home Confinement during the stringent lockdown	
	Confined (vs. Not confined) (*n* = 1,458)	
	Effect Estimate^b^	CI Limit^c^
Subjective well-being		
Life satisfaction	1.45	1.12
Happiness	1.45	1.00
Meaning	1.42	1.00
Social well-being		
Social support	1.00	1.00
Loneliness	1.47	1.05
Prosocial/altruistic behavior		
Volunteering	2.50	1.00
Caring	1.63	1.00
Compliance with COVID-19 rules	1.93	1.62
Psychological distress		
Depressive symptoms	1.50	1.12
Anxiety	1.47	1.16
Number of minor stressors	1.56	1.27
Number of major stressors	1.77	1.40
Thoughts of self-harm	1.46	1.00
Health behaviors		
No unhealthy change in smoking	2.15	1.00
No unhealthy change in alcohol drinking	1.00	1.00
No unhealthy change in diet	1.11	1.00
Gentle physical activity	2.84	2.30
High intensity physical activity	3.87	2.04
Exercising at home	1.49	1.00
Good sleep	1.00	1.00

Abbreviations: CI, confidence interval

^a^ See VanderWeele and Ding (2017) for the formula for calculating E-values

^b^ E-values for effect estimates are the minimum strength of association on the risk ratio scale that an unmeasured confounder would need to have with both the exposure and the outcome, above and beyond the measured covariates, to fully explain away the observed association of home confinement during the stringent lockdown (reference: “Not confined”) with the outcomes

^c^ E-values for the 95% CI limit closest to the null denote the minimum strength of association on the risk ratio scale that an unmeasured confounder would need to have with both the exposure and the outcome, above and beyond the measured covariates, to shift the 95% CI to include the null value

## Discussion

The strict lockdown enacted during the first wave of COVID-19 in the UK mandated people to avoid nonessential travel and physical contact with others. By stringently adhering to the stay-at-home directives, people limited their risk of SARS-CoV-2 infection and supported the broader public health response to COVID-19. However, it remains unclear whether “staying at home” during the initial lockdown in the UK had longer-term implications for individual health and well-being. We used longitudinal data from a cohort of adults to estimate the effects of home confinement during the initial lockdown in the UK on a wide range of health and well-being outcomes assessed 8 weeks later after homebound restrictions had been eased. Our main findings are four-fold. First, at baseline, participants who remained at home tended to be from lower socioeconomic backgrounds (e.g., lower education, low income). Second, home confinement was associated with greater subsequent compliance with COVID-19 rules, more perceived major stressors, and a lower prevalence of physical activity post-lockdown. Third, there was modest evidence that home confinement was associated with lower life satisfaction, greater loneliness, greater depressive symptoms, greater anxiety symptoms, and more perceived minor stressors post-lockdown. Fourth, there was little evidence that home confinement was associated with other indices of subsequent health and well-being.

This longitudinal study's general pattern of findings is consistent with previous (mostly cross-sectional) research that suggests homebound orders have had negative consequences for individual health and well-being. For example, studies have linked stay-at-home regulations with higher levels of psychological distress and lower physical activity levels (Ammar et al., 2020; Hermassi et al., 2021). The consistency in findings across study designs (cross-sectional versus longitudinal) might indicate that bias due to reverse causation, which we partly addressed by using longitudinal data, was not large enough to change the conclusions of the analyses qualitatively. However, this study adds to the existing evidence because it is one of the first to measure and estimate the effects of *actual* self-reported home confinement behavior during a lockdown on subsequent health and well-being. In contrast with many studies that have inferred home confinement via population-level lockdown regulations enacted in a particular context and time (Amanzio et al., 2021; Simor et al., 2021), our results indicate how home confinement behavior of individuals during lockdown conditions might affect their own functioning. Ultimately, lockdown conditions restrict people from accessing valued resources in the outside environment (e.g., workplaces, places of worship) that play a role in supporting well-being. Disrupted access to valued resources may have degraded aspects of health and well-being to a greater extent among people who were confined to their homes. Notably, the distribution of home confinement during the lockdown was socially patterned—for example, low-income and less educated individuals reported higher home confinement behavior at baseline—and suggests that home confinement may have amplified existing health disparities. Although the reason for such social patterning in home confinement remains unclear and is worth further investigation in future studies, the individuals from lower socioeconomic backgrounds might have had less green space to go to (e.g., no gardens or fewer parks nearby) and had fewer supports with childcare. Moreover, adults from higher socioeconomic backgrounds might be more aware of the need to maintain daily exercise during the pandemic.

Whereas prior studies in this area have typically examined a single or narrow set of outcomes focused on a specific domain of human life at a time (Ammar et al., 2020; Fernandez-Rio et al., 2020), our study provided more holistic evidence for potential impacts of home confinement on subsequent health and wellbeing by examining a wide range of outcomes simultaneously. First, we found some evidence that home confinement was associated with at least one index of psychological distress, subjective well-being (life evaluation), social well-being (loneliness), prosocial/altruistic behavior (compliance with COVID-19 rules), and health behaviors (physical activity), suggesting home confinement may have some implications for different domains of health and wellbeing. Second, there was variability in the estimated effects within domains of outcomes. For example, home confinement was associated with a lower prevalence of gentle and high-intensity physical activity, but there was little evidence of association with the other health behavior outcomes that we examined. In contrast, home confinement was associated with most outcomes within the domain of psychological distress, suggesting that home confinement may have a particularly pervasive impact on mental well-being.

While there was evidence for persistent impacts of home confinement on some health and wellbeing outcomes post-lockdown, there was no evidence of association with home confinement for 11 of the 20 outcomes. Drawing on Conservation of Resources (COR) theory (Hobfoll et al., 2016; Holmgreen et al., 2017), it is possible that, as the lockdown conditions eased, people who were confined to their homes during the lockdown were able to incrementally gain more resources (e.g., social support, coping skills), which could have contributed to the recovery of aspects of health and well-being over time (Counted et al., 2021). An alternative explanation is that confined individuals adapted psychologically over time and regained homeostasis as they adjusted to their circumstances. This theorizing resonates with the notion of resilience, which can be understood as an interactive process in which an individual is able to adapt successfully to adversity by harnessing resources (e.g., environmental, psychosocial) that support well-being (Southwick et al., 2014). Despite facing COVID-related stressors, participants who were confined to their homes may have been able to develop, access, or acquire resilience resources that enabled them to rebound from home confinement over time. This perspective is supported by the results that emerged when the outcomes were taken from week 20, which revealed that the observed associations for most outcomes shifted closer to the null when compared to 8 weeks earlier.

### Strengths and Limitations

Key strengths of this study include: 1) direct assessment of home confinement during the UK lockdown, 2) leveraging panel data and rigorously adjusting for pre-baseline covariates including a proxy of prior exposure values to account for confounding and some reverse causation, and 3) the outcome-wide analytic approach assessing potential effects of home confinement on a range of health and well-being outcomes simultaneously. However, the strengths of this study should be considered alongside its limitations. First, the sample comprised UK adults who were not representative of the national population. Our analytic approach included survey weights to improve the generalizability of the findings to the wider UK population. However, the transportability of the findings to populations living in other contexts may be limited. Second, the findings of this study are limited by our use of self-report data. Future research could yield further insights into the effects of place confinement by using multiple methods and/or informants to assess outcomes of interest more comprehensively. Third, with observational data, there is a possibility that estimated effects may be biased due to unmeasured confounding. We adjusted for numerous pre-baseline covariates, including pre-baseline values of social isolation status as a proxy of home confinement. In addition, E-values suggested that some of the observed associations might be moderately robust to potential unmeasured confounding. However, we could not adjust for pre-pandemic values of the outcomes as the data were not available. We cannot completely rule out the possibility that one or more of the observed associations could be explained away by unmeasured confounding and reverse causation.

## Conclusion

This study is one of the first longitudinal studies to estimate the effects of place confinement on individual health and well-being (both within the context of the COVID-19 pandemic and more generally). We found some evidence indicating that home confinement during the stringent initial COVID-19 pandemic lockdown in the UK was persistently associated with selected indices of health and well-being—particularly psychological distress—even after the lockdown has ended. Notwithstanding the need for additional research to replicate and expand on the results reported herein, our findings suggest that interventions and public health initiatives which help to alleviate loneliness, encourage healthy behaviors, and reduce psychological distress could support people as they deal with the challenges of being emplaced and perhaps contribute to accelerating post-pandemic recovery after lockdowns have ended.

## Supplementary Information

Below is the link to the electronic supplementary material.

Supplementary file1 (DOCX 69 KB)
