# Supplementary material for: Associations of home confinement during COVID-19 lockdown with subsequent health and well-being among UK adults
Source: Curr Psychol. 2022 Mar 15:1–10. Online ahead of print. doi: 10.1007/s12144-022-03001-5 (PMC8922081; doi:10.1007/s12144-022-03001-5)
Supplement: Supplementary file 1 — Supplementary file1 (DOCX 69 KB) [file 12144_2022_3001_MOESM1_ESM.docx]

Supplementary Table S1. List of Health and Well-being Outcomes.

| Outcomes by Theme | Variable Name in UCL COVID-19 Social Study | Variable Type | Item Question/Scale Name |
| --- | --- | --- | --- |
| 1. Subjective well-being | | |  |
| Life satisfaction | onssat | Continuous (range: 0-10); higher values indicate greater life satisfaction. | Overall, in the past week, how satisfied have you been with your life? |
| Happiness | onshappy | Continuous (range: 0-10); higher values indicate greater happiness. | In the past week, how happy did you feel? |
| Meaning | onsworth | Continuous (range: 0-10); higher values indicate greater sense of meaning. | In the past week, to what extent have you felt the things you are doing in your life are worthwhile? |
| 2. Social well-being | | |  |
| Social support | support | Continuous (range: 1-30); higher values indicate more support. | Brief Perceived Social Support Questionnaire (F-SozU K-6) |
| Loneliness | lonely | Continuous (range: 1-9); higher values indicate greater loneliness. | UCLA 3-item Loneliness Scale |
| 3. Character and virtue | | |  |
| Volunteering | acta8 | Binary (0 = Did not do vs 1 = <30 mins/30 mins-2 hours/3-5 hours/6-+ hours) | How long you’ve spent doing different activities on a single day (the last week day): Volunteering |
| Caring | acta7 | Binary (0 = Did not do vs 1 = <30 mins/30 mins-2 hours/3-5 hours/6-+ hours) | How long you’ve spent doing different activities on a single day (the last week day): Caring for a friend or relative |
| Compliance with COVID-19 rules | followingisolation | Continuous (range: 1-7); higher values indicate greater compliance. | Are you following the recommendations from authorities to prevent spread of Covid-19? |
| 4. Psychological distress | | |  |
| Depressive Symptoms | PHQ | Continuous (range:0-27); higher values indicate more depressive symptoms. | Patient Health Questionnaire-9 |
| Anxiety | GAD | Continuous (range: 0-21); higher values indicate more anxiety. | Generalized Anxiety Disorder-7 |
| Number of minor stressors | stressorsminor___1~  stressorsminor___16 | Continuous (total number of stressors; range: 0 -16) | Over the past week, have any of the following been worrying you at all, even if only in a minor way? |
| Number of major stressors | stressorsmajor___1~  stressorsmajor___16 | Continuous (total number of stressors; range: 0 -16) | Have any of these things been causing you SIGNIFICANT stress? (e.g. they have been constantly on your mind or have been keeping you awake at night) |
| Thoughts of self-harm | harm1 | Binary (0 = Not at all vs 1 = One or two days/more than half the days/nearly every day) | Over the last week, how often have been bothered by: Self-harming or deliberately hurting yourself |
| 5. Health behavior | | |  |
| No unhealthy change in smoking | smokechange | Binary (0 = Less than usual/About the same/I don’t smoke vs 1 = More than usual) | Over the past week have you smoked more than usual? |
| No unhealthy change in alcohol drinking | alcoholchange | Binary (0 = Less than usual/About the same/I don’t drink alcohol vs 1 = More than usual) | Over the past week have you drunk alcohol more than usual? |
| No unhealthy change in diet | dietchange_3 | Binary (0 = About the same healthiness as usual/more healthy than usual vs 1 = Less healthy than usual) | Over the past week how has your diet been? |
| Gentle physical activity | actb3 | Binary (0 = Did not do vs 1 = <30 mins/30 mins-2 hours/3-5 hours/6-+ hours) | How long you’ve spent doing different activities on a single day (the last week day): Going out for a walk or other gentle physical activity |
| High intensity physical activity | actb4 | Binary (0 = Did not do vs 1 = <30 mins/30 mins-2 hours/3-5 hours/6-+ hours) | How long you’ve spent doing different activities on a single day (the last week day): Going out for moderate or high intensity activity (e.g, running, cycling or swimming) |
| Exercising at home | actb6 | Binary (0 = Did not do vs 1 = <30 mins/30 mins-2 hours/3-5 hours/6-+ hours) | How long you’ve spent doing different activities on a single day (the last week day): Exercising inside your home or garden (e.g. doing yoga, weights or indoor exercise) |
| Good sleep | sleep | Binary (0 = Average/Not good/Very poor vs 1 = Very good/Good) | Over the past week, how has your sleep been? |

Supplementary Table S2. Number of Missing Data in the Analytic Sample (n = 10,630)

| Characteristics at the beginning of lockdown |  |  |  |  |
| --- | --- | --- | --- | --- |
|  | Week 1 | | Week 12 | |
|  | N of missing | % of missing | N of missing | % of missing |
| **Sociodemographic factors** |  |  |  |  |
| Age, years | 0 | 0 | NA | NA |
| Female gender | 43 | 0.4 | NA | NA |
| Non-white ethnicity | 26 | 0.2 | NA | NA |
| Living alone | 0 | 0.0 | NA | NA |
| Education | 0 | 0.0 | NA | NA |
| Employed | 0 | 0.0 | NA | NA |
| Any key worker role | 0 | 0.0 | NA | NA |
| Low Income (<£30 000) | 1099 | 10.3 | NA | NA |
| **Physical health and health behaviors** |  |  |  |  |
| Number of health conditions | 544 | 5.1 | NA | NA |
| Smoking status | 0 | 0.0 | NA | NA |
| Number of alcoholic drinks in the past week | 0 | 0.0 | NA | NA |
| **Pre-pandemic service attendance** | 1499 | 14.1 | NA | NA |
| **Staying at home** | 0 | 0 | NA | NA |
| **Social relationships** |  |  |  |  |
| Meeting up with people in usual life | 0 | 0 | NA | NA |
| Number of close friends | 0 | 0 | NA | NA |
| **Personality** |  |  |  |  |
| Neuroticism (range: 3-21) | 0 | 0 | NA | NA |
| Extraversion (range: 3-21) | 0 | 0 | NA | NA |
| Openness (range: 3-21) | 0 | 0 | NA | NA |
| Agreeableness (range: 3-21) | 0 | 0 | NA | NA |
| Conscientiousness (range: 3-21) | 0 | 0 | NA | NA |
| Outcome variables | Week 1 | | Week 12 | |
|  | N of missing | % of missing | N of missing | % of missing |
| **Subjective well-being** |  |  |  |  |
| Life satisfaction | 80 | 0.8 | 15 | 0.14 |
| Happiness | NA | NA | 15 | 0.14 |
| Meaning | 80 | 0.8 | 15 | 0.14 |
| **Social well-being** |  |  |  |  |
| Social support | 195 | 1.8 | 73 | 0.69 |
| Loneliness | 194 | 1.8 | 73 | 0.69 |
| **Prosocial/altruistic behavior** |  |  |  |  |
| Volunteering | 385 | 3.6 | 169 | 1.59 |
| Caring | 295 | 2.8 | 117 | 1.10 |
| Compliance with COVID-19 rules | 2 | 0.0 | 34 | 0.32 |
| **Psychological distress** |  |  |  |  |
| Depressive symptoms | 113 | 1.1 | 55 | 0.52 |
| Anxiety | 131 | 1.2 | 66 | 0.62 |
| Number of minor stressors | 0 | 0.0 | 0 | 0.00 |
| Number of major stressors | 0 | 0.0 | 0 | 0.00 |
| Thoughts of self-harm | NA | NA | 81 | 0.76 |
| **Health behaviors** |  |  |  |  |
| No unhealthy change in smoking | 118 | 1.1 | 41 | 0.39 |
| No unhealthy change in alcohol drinking | 107 | 1.0 | 48 | 0.45 |
| No unhealthy change in diet | NA | NA | 47 | 0.44 |
| Gentle physical activity | 314 | 3.0 | 145 | 1.36 |
| High intensity physical activity | 391 | 3.7 | 132 | 1.24 |
| Exercising at home | 312 | 2.9 | 131 | 1.23 |
| Good sleep | 0 | 0.0 | 0 | 0.00 |

Supplementary Table S3. Baseline Characteristics From Week 1 Most Strongly Associated With the Health and Well-being Outcomes From Week 12, COVID-19 Social Study (n = 10,630).^a^

| Outcomes in week 20 | Baseline characteristics from week 1 most strongly associated with the outcomes^b^ | | | | | |
| --- | --- | --- | --- | --- | --- | --- |
|  | Top 1 | | Top 2 | | Top 3 | |
|  | Variable | Est.^c^ | Variable | Est.^c^ | Variable | Est.^c^ |
| Subjective well-being |  |  |  |  |  |  |
| Life satisfaction | Living alone | 1.27 | Smoking status: Non-smoker (vs. current smoker) | 1.21 | Pre-pandemic service attendance: Less than once a week (vs. at least once a week) | 1.16 |
| Happiness | Living alone | 1.30 | Smoking status: Non-smoker (vs. current smoker) | 1.24 | Number of health conditions | 1.13 |
| Meaning | Living alone | 1.27 | Smoking status: Non-smoker (vs. current smoker) | 1.24 | Pre-pandemic service attendance: Less than once a week (vs. at least once a week) | 1.15 |
| Social well-being |  |  |  |  |  |  |
| Social support | Living alone | 2.09 | Low income | 1.30 | Meeting up with people in usual life: Less than once a week (vs. every day) | 1.18 |
| Loneliness | Living alone | 1.53 | Smoking status: Non-smoker (vs. current smoker) | 1.23 | Smoking status: Ex-smoker (vs. current smoker) | 1.17 |
| Prosocial/altruistic behavior |  |  |  |  |  |  |
| Volunteering | Meeting up with people in usual life: Less than once a week (vs. every day) | 2.51 | Pre-pandemic service attendance: Not at all (vs. at least once a week) | 1.90 | Meeting up with people in usual life: Once a week or more often (vs. every day) | 1.80 |
| Caring | Living alone | 2.27 | Education: Degree or above (vs. GCSE or below) | 2.12 | Meeting up with people in usual life: Less than once a week (vs. every day) | 1.38 |
| Compliance with COVID-19 rules | Meeting up with people in usual life: Less than once a week (vs. every day) | 1.23 | Pre-pandemic service attendance: Less than once a week (vs. at least once a week) | 1.17 | Meeting up with people in usual life: Once a week or more often (vs. every day) | 1.14 |
| Psychological distress |  |  |  |  |  |  |
| Depressive symptoms | Smoking status: Non-smoker (vs. current smoker) | 1.27 | Number of health conditions | 1.26 | Living alone | 1.19 |
| Anxiety | Smoking status: Non-smoker (vs. current smoker) | 1.22 | Number of health conditions | 1.17 | Pre-pandemic service attendance: Less than once a week (vs. at least once a week) | 1.15 |
| Number of minor stressors | Smoking status: Non-smoker (vs. current smoker) | 1.19 | Pre-pandemic service attendance: Less than once a week (vs. at least once a week) | 1.18 | Non-white ethnicity | 1.14 |
| Number of major stressors | Non-white ethnicity | 1.27 | Smoking status: Non-smoker (vs. current smoker) | 1.26 | Smoking status: Ex-smoker (vs. current smoker) | 1.19 |
| Thoughts of self-harm | Pre-pandemic service attendance: Less than once a week (vs. at least once a week) | 5.16 | Pre-pandemic service attendance: Not at all (vs. at least once a week) | 3.67 | Smoking status: Ex-smoker (vs. current smoker) | 2.77 |
| Health behaviors |  |  |  |  |  |  |
| No unhealthy change in smoking | Smoking status: Non-smoker (vs. current smoker) | 115.58 | Smoking status: Ex-smoker (vs. current smoker) | 23.10 | Meeting up with people in usual life: Less than once a week (vs. every day) | 1.93 |
| No unhealthy change in alcohol drinking | Pre-pandemic service attendance: Less than once a week (vs. at least once a week) | 1.05 | Any key worker role | 1.04 | Pre-pandemic service attendance: Not at all (vs. at least once a week) | 1.03 |
| No unhealthy change in diet | Female gender | 1.09 | Living alone | 1.07 | Smoking status: Non-smoker (vs. current smoker) | 1.06 |
| Gentle physical activity | Meeting up with people in usual life: Less than once a week (vs. every day) | 1.14 | Female gender | 1.11 | Meeting up with people in usual life: Once a week or more often (vs. every day) | 1.09 |
| High intensity physical activity | Female gender | 1.60 | Smoking status: Non-smoker (vs. current smoker) | 1.51 | Smoking status: Ex-smoker (vs. current smoker) | 1.45 |
| Exercising at home | Pre-pandemic service attendance: Less than once a week (vs. at least once a week) | 1.55 | Smoking status: Non-smoker (vs. current smoker) | 1.52 | Smoking status: Ex-smoker (vs. current smoker) | 1.49 |
| Good sleep | Smoking status: Non-smoker (vs. current smoker) | 1.38 | Smoking status: Ex-smoker (vs. current smoker) | 1.22 | Non-white ethnicity | 1.21 |

^a^ Home confinement during the stringent lockdown (March 23 - May 13, 2020) was assessed in week 4 (April 11 - April 17, 2020). Outcomes were assessed in week 12 (June 6 - June 12, 2020). Covariates were measured at the beginning of the lockdown (week 1, March 21 - March 27, 2020). The analytic sample was restricted to those who had participated in the survey in both week 1 and week 12. Multiple imputation was performed to impute missing data on the covariates and the outcomes.

^b^ All models were controlled for pre-baseline participants’ characteristics from week 1, including sociodemographic characteristics (age, gender, race, living alone, education, employment, any key worker role, and low income), health conditions and health behaviors (number of health conditions, current smoking, and number of alcohol drinks in the past week, current smoking status, and number of alcoholic drinks in the past week), pre-pandemic religious service attendance, social relationships (frequency of meeting up with people in usual life and number of close friends), personality (neuroticism, extraversion, openness, agreeableness, and conscientiousness), and the pre-baseline exposure level (home confinement at week 1). Data were weighted to the proportions of gender, age, ethnicity, education, and country of living obtained from the Office for National Statistics

^c^ Estimates are on the risk ratio scale. For the continuous outcomes (life satisfaction, happiness, meaning, social support, loneliness, compliance with COVID-19 rules, depressive symptoms, anxiety, and number of minor and major stressors), we used the conversion outlined in VanderWeele and Ding (2017) to get an approximated risk ratio from a regression coefficient. For the rare binary outcomes (volunteering, thoughts of self-harm, no unhealthy change in smoking behaviors, and high intensity physical activity), the estimated odds ratios approximate risk ratios.

Supplementary Figure S1. Sensitivity Analysis Comparing Associations Between Home Confinement in Week 4 and Subsequent Continuous Indicators of Health and Well-being in the UK, COVID-19 Social Study (n = 10,630).

* p<0.05 before Bonferroni correction; ** p<0.01 before Bonferroni correction; *** p<0.05 after Bonferroni correction (the p-value cutoff for Bonferroni correction is p = 0.05/20 outcomes = p <0.0025).

Home confinement during the stringent lockdown (March 23 - May 13, 2020) was assessed in week 4 (April 11 - April 17, 2020). Covariates were measured at the beginning of the lockdown (week 1, March 21 - March 27, 2020). Outcomes were assessed either in week 12 (June 6 - June 12, 2020) or in week 20 (August 1 - August 7, 2020). The analytic samples were restricted to those who had participated in the survey in both week 1 and week 12 or week 20. Multiple imputation was performed to impute missing data on the covariates and the outcomes. All continuous outcomes (life satisfaction, happiness, meaning, social support, loneliness, compliance with COVID-19 rules, depressive symptoms, anxiety, and number of minor and major stressors) were standardized (mean = 0, standard deviation, 1), and β was the standardized effect size. All models were controlled for pre-baseline participants’ characteristics from week 1, including sociodemographic characteristics (age, gender, race, living alone, education, employment, any key worker role for the analyses not excluding key workers, and low income), health conditions and health behaviors (number of health conditions, current smoking, and number of alcohol drinks in the past week, current smoking status, and number of alcoholic drinks in the past week), pre-pandemic religious service attendance, social relationships (frequency of meeting up with people in usual life and number of close friends), personality (neuroticism, extraversion, openness, agreeableness, and conscientiousness), and the pre-baseline exposure level (home confinement at week 1). Data were weighted to the proportions of gender, age, ethnicity, education, and country of living obtained from the Office for National Statistics.

Supplementary Figure S2. Sensitivity Analysis Comparing Associations Between Home Confinement in Week 4 and Subsequent Binary Indicators of Health and Well-being in the UK, COVID-19 Social Study (n = 10,630).

* p<0.05 before Bonferroni correction; ** p<0.01 before Bonferroni correction; *** p<0.05 after Bonferroni correction (the p-value cutoff for Bonferroni correction is p = 0.05/20 outcomes = p <0.0025)

Home confinement during the stringent lockdown (March 23 - May 13, 2020) was assessed in week 4 (April 11 - April 17, 2020). Covariates were measured at the beginning of the lockdown (week 1, March 21 - March 27, 2020). Outcomes were assessed either in week 12 (June 6 - June 12, 2020) or in week 20 (August 1 - August 7, 2020). The analytic samples were restricted to those who had participated in the survey in both week 1 and week 12 or week 20. Multiple imputation was performed to impute missing data on the covariates and the outcomes. The estimates for the outcomes of volunteering, thoughts of self-harm, no unhealthy change in smoking behaviors, and high intensity physical activity were odds ratios estimated via weighted logistic regression; these outcomes were rare (prevalence <10%), so the odds ratios would approximate the risk ratios. The estimates for other nonrare, dichotomized outcomes (caring, no unhealthy change in drinking, no unhealthy change in diet, gentle physical activity, exercising at home, and good sleep) were risk ratios estimated via weighted Poisson regression. All models were controlled for pre-baseline participants’ characteristics from week 1, including sociodemographic characteristics (age, gender, race, living alone, education, employment, any key worker role for the analyses not excluding key workers, and low income), health conditions and health behaviors (number of health conditions, current smoking, and number of alcohol drinks in the past week, current smoking status, and number of alcoholic drinks in the past week), pre-pandemic religious service attendance, social relationships (frequency of meeting up with people in usual life and number of close friends), personality (neuroticism, extraversion, openness, agreeableness, and conscientiousness), and the pre-baseline exposure level (home confinement at week 1). Data were weighted to the proportions of gender, age, ethnicity, education, and country of living obtained from the Office for National Statistics.
